# Effects of Storage Conditions on the Flavor Stability of Fried Pepper (*Zanthoxylum bungeanum*) Oil

**DOI:** 10.3390/foods10061292

**Published:** 2021-06-04

**Authors:** Jie Sun, Baoguo Sun, Fazheng Ren, Haitao Chen, Ning Zhang, Yuyu Zhang, Huiying Zhang

**Affiliations:** 1 Beijing Key Laboratory of Flavor Chemistry, Beijing Technology and Business University, Beijing 100048, China; sunjeel@163.com (J.S.); sunbg@btbu.edu.cn (B.S.); zh_ningts@btbu.edu.cn (N.Z.); zhangyuyu@btbu.edu.cn (Y.Z.); zhanghuiying@btbu.edu.cn (H.Z.); 2 Beijing Advanced Innovation Center for Food Nutrition and Human Health, College of Food Science & Nutritional Engineering, China Agricultural University, Beijing 100083, China; renfazheng@cau.edu.cn

**Keywords:** fried pepper oil, ultraviolet (UV) irradiation, oxygen exposure, storage, aroma attenuation

## Abstract

Flavor stability of fried pepper oil was investigated during 30 days of storage. Variation trends of key volatile flavor compounds in fried pepper oil induced by ultraviolet (UV) irradiation and oxygen (O_2_) exposure were compared using GC-MS and chiral GC-MS analysis. Chirality analysis showed that conversion of (S)-(-)-limonene to (R)-(+)-limonene form was observed during storage. The storage conditions did not change the configuration of linalool, linalool oxide, or carvone. Quantitative analysis showed that the concentrations of linalool, limonene, 1,8-cineole, β-myrcene, and β-ocimene decreased dramatically during storage, whereas carvone, (E)-2-heptenal, and linalool oxide showed an increasing trend during storage. The loss rate of limonene and linalool exhibited the highest under combined UV and O_2_ condition, which played an important role for the aroma attenuation of pepper oil. This result will benefit the storage of pepper oil and based on pepper oil aromatic products.

## 1. Introduction

*Zanthoxylum bungeanum Maxim*. belongs to the *Zanthoxylum* genus in Ruratceae and is estimated to have 250 species throughout the world [1]. In China, it is also known as Chinese pepper or huajiao. With the deepening of targeted poverty alleviation efforts, Chinese pepper, as an efficient agricultural product for farming, has been widely planted across the country. In recent years, the total production of Chinese pepper has shown a fluctuating growth trend. According to statistics from the China Forestry Yearbook, the output of dried Chinese pepper has remained above 300,000 tons since 2012. Production increased to 438,400 tons in 2017 and to approximately 458,500 tons in 2018. Because of its dual medicinal and edible purposes, it has become one of the hotspots of research by scientific researchers and manufacturing companies.

*Zanthoxylum bungeanum Maxim*. is popular among consumers because of its unique and mouth-watering aroma and taste. The composition of *Zanthoxylum bungeanum Maxim.* volatiles has been studied in depth and found to be mainly composed of monoterpenes [2,3,4]. Jiang et al. [5] reported that geraniol, citronellal, linalool, and methyl cinnamate were perceived to be important to the basic flavor of green and ripe fruits and dried pericarp of Japanese pepper. Yang et al. [6] compared the volatile flavor compounds of Sichuan red pepper and green pepper, and found that linalool, α-terpineol, myrcene, and 1,8-cineole were the main aroma compounds of the two types of pepper. As the main deep-processed product of Chinese pepper, fried pepper oil is widely used in various foods. Our previous research on the flavor compounds of fried pepper oil showed that 1,8-cineole, (E)-2-heptenal, β-myrcene, β-ocimene, limonene, and linalool were the major aroma active compounds in pepper oil from two regions (Hancheng and Hanyuan) through solvent-assisted flavor evaporation combined with gas chromatography-mass spectrometry and aroma extract dilution analysis [7].

However, the flavor intensity of fried pepper oil could decay after a period of storage when it was kept under sunlight at room temperature. Fried pepper oil contained a large number of terpenoids, and studies have shown that terpenoids were very susceptible to oxygen and ultraviolet radiation [8]. As long as there was oxygen, even at a low concentration, terpenoids could undergo oxidation reactions. The resulting oxidation products would be further degraded due to unstable structure and produce volatile components such as alcohols, aldehydes, and ketones [8]. In addition, previous studies have shown that storage conditions (such as storage time, temperature, light, and oxygen) may have a significant impact on the aroma of essential oils. Sun et al. found the pummelo essential oil could deteriorate very quickly with a strong oily off-flavor, for which UV sunlight was revealed to be the critical contributor causing the chemical reactions for the aroma changes [9]. Cheng et al. [10] investigated the changes of limonene and linalool in pepper flavoring oil under different storage conditions and found the contents of limonene and linalool were significantly reduced under sunlight irradiation. Moreover, due to the influence of factors such as pH and temperature, many chiral molecules in terpenoids can be transformed from one form to another, thereby changing the aroma quality of the products [11]. It has been reported that both (S)-a-terpineol and (S)-4-terpineol in the hard tea beverage dominated at beginning of the storage, but (R)-(+)-a-terpineol became dominated after storage; thus, racemization affected the flavor stability of the hard tea beverage [12]. Therefore, the changes of terpenoids under different storage conditions were crucial to the aromatic profiles of fried pepper oil. 

Despite all this, studies on the effects of UV irradiation and oxygen exposure on the aromatic changes of fried pepper oil are still limited. In the previous study, our research team reported that 1,8-cineole, (E)-2-heptenal, β-myrcene, β-ocimene, limonene, linalool, linalool oxide, and carvone were the key volatile flavor compounds in fried pepper oil [7]. Therefore, the objective of the study was to analyze the effect of UV irradiation and oxygen exposure on above-mentioned volatile flavor compounds and elucidate the key volatiles responsible for the aroma changes after UV irradiation and oxygen exposure.

## 2. Materials and Methods

### 2.1. Materials

Dried Hanyuan (HY) pepper samples were purchased from You Jia Co., Ltd. (Yaan City, Sichuan Province, China). Fresh peppers were harvested in September 2020 and dried by hot air. Corn germ oil was purchased from Jin Long Yu Co., Ltd. (Qinhuangdao City, Hebei Province, China).

### 2.2. Chemicals

Limonene (≥95%), β-ocimene (95%), (E)-2-heptenal (95%), 1,8-cineole (99%), carvone (≥95%), β-myrcene (95%), linalool (98%), linalool oxide (97%), and n-alkane (C6-C30, ≥99%) standards and the internal standard 4-octanol (≥99%) were purchased from Sigma-Aldrich (Shanghai, China). Dichloromethane and sodium sulfate were purchased from Thermo Fisher (Beijing, China). Enantiomer standards including S-(-)-limonene (96%), R-(+)-limonene (97%), R-(-)-linalool (97%), R-(-)-carvone (97%), and S-(+)-carvone (97%) were purchased from Sigma Aldrich (Shanghai, China).

### 2.3. Fried Pepper Oil Sample Preparation

Corn germ oil (150 g) was heated to 130 °C in an oil bath. Then, dry pepper granules (25 g) were added to the corn germ oil and fried at 130 °C continuously for 20 min. During frying, the mixture was constantly stirred to prevent local overheating. The pepper oil was cooled to room temperature using an ice-water bath, and the pepper granules were removed. The prepared pepper oil was immediately subjected to a storage experiment.

### 2.4. Pepper Oil with Different Storage Conditions

The effects of UV irradiation and oxygen exposure on the aroma quality of pepper oil were investigated at room temperature. The experimental samples were divided into four groups with label numbers 1–4. The sample (with three replicates each) for each storage time (each two days) was measured for a total of 30 days. Detailed sample preparation was as follows:

(1) The No. 1 sample was only treated with UV irradiation. The pepper oil (100 g) sample was stored in a transparent and sealed glass bottle, and nitrogen (99.9%) was filled into the bottle to discharge the air. Then, the glass bottles were placed in an incubator and exposed under four ultraviolet lights with peak emission at 254 nm at a distance of 10 cm for 30 days under a power of 10 W. The temperature of the incubator was controlled at 25 °C.
(2) The No. 2 sample was only treated with oxygen exposure. The pepper oil (100 g) sample was stored in a transparent and sealed glass bottle with wrapped aluminum foil. Then, the glass bottles were placed in an incubator at 25 °C. Oxygen was injected into the glass bottles every 2 days.
(3) The No. 3 sample that was simultaneously treated with UV irradiation and oxygen exposure. The subsequent procedures followed those for sample 1. Oxygen was injected into the glass bottles every 2 days.
(4) The No. 4 sample (newly prepared pepper oil) was directly analyzed.

### 2.5. Separation of Volatile Compounds

Pepper oil (50 g) was dissolved in dichloromethane (200 mL) by shaking at 120 rpm (grant OLS200, Cambridgeshire, UK) for 0.5 h. Additionally, 4-octanol with a concentration of 8.2 mg/L was added as internal standard before the extraction procedure. The solutions were then subjected to high-vacuum distillation using SAFE. The resulting distillate was dried over anhydrous Na_2_SO_4_ and filtered. The final distillate was concentrated to 6–8 mL using a Vigreux column (50 × 1 cm) (Beijing Jingxing Glassware Co., Ltd., Beijing, China) and then further concentrated to 1 mL with a nitrogen blower.

### 2.6. GC-MS Analysis and Chiral GC-MS Analysis

The analysis methods were performed according to Sun et al. [7]. GC-MS analysis was performed by a Thermo Fisher Trace 1310 gas chromatograph (Thermo Fisher Scientific, Waltham, MA, USA) coupled with a Thermo Fisher mass spectrometer (Thermo Fisher Scientific). Separation was performed with TG-Wax (30 m × 0.25 mm i.d., 0.25 μm, Thermo Fisher Scientific). The oven temperature was initially 40 °C, followed by a 1 min hold; increased to 140 °C at a rate of 2 °C/min, followed by a 1 min hold; and finally increased to 220 °C at a rate of 6 °C/min, followed by a 1 min hold. A Trace GC-MS system was used for chiral GC-MS analysis. The GC system was equipped with a 2,3-dimethyl-6-tert-butyldimethylchlorosilane-β-cyclodextrin capillary column (30 m × 0.25 mm i.d., 0.25 μm) from BGB (Switzerland). The mass detector conditions were as follows: ionization energy, 70 eV; ion source temperature, 250 °C; mass range, *m*/*z* 35–300; and solvent delay, 5 min.

### 2.7. Identification and Quantitation

Volatile flavor compounds were identified by comparing their retention indices (RIs), mass spectra, and standard reference compounds. The correction concentrations of each aroma compound were calculated according to the 4-octanol internal standard.

### 2.8. Loss or Grow Rate of Key Volatile Compounds

The loss rate or grow rate of key flavor compounds in fried pepper oil was calculated as follows:

(1) $$p=\frac{m_{1}-m_{2}}{m_{1}}$$

where *p* represents the loss rate or grow rate of key flavor compounds, *m*_1_ is the concentration of key aroma compounds in the control pepper oil sample, and *m*_2_ is the concentration of key aroma compounds in the pepper oil sample stored for 30 days.

### 2.9. Statistical Analysis

The experimental results were expressed as the mean ± standard deviation (SD) and analyzed by one-way analysis of variance (ANOVA) using SPSS-IBM 19.0 software. Tukey’s post hoc test (*p* < 0.05) was performed to compare significantly different means and samples. The figures were drawn using Origin 2018 software (OriginLab, Northampton, MA, USA).

## 3. Results and Discussion

### 3.1. Effects of Different Storage Conditions on Enantiomer Ratios of Chiral Compounds

It has been confirmed that the enantiomer of a chiral compound usually has different flavor characteristics and odor threshold [13]. Enantiomeric composition of limonene, linalool, linalool oxide, and carvone were investigated in this study by chiral GC-MS and by using the retention times of authentic reference standards as well as mass spectra (Table 1). The results showed that S-(-)-limonene, S-(+)-linalool, R-(-)-carvone, and 2R, 5R-linalool oxide were the major isomers, respectively, in newly prepared fried pepper oil. Li et al. reported that a conversion of (R)-(-)-linalool to (S)-(+) form in the hard tea beverage was observed at a higher temperature during storage [12]. Therefore, changes in the enantiomeric ratio of key compounds in pepper oil under different storage conditions could affect the overall flavor of pepper oil.

The enantiomeric composition changes of limonene, linalool, linalool oxide, and carvone under different storage conditions were listed in Table 2. In 30 days of storage, (S)/(R) ratio of limonene decreased due to the conversion of S-limonene to R-limonene when treated solely with UV irradiation or oxygen exposure. Under UV irradiation and oxygen exposure conditions, an increase of R-limonene and decrease of S-limonene were observed. At 20 days of storage, S-limonene and R-limonene accounted for the same ratio, and R-limonene began to dominate after 20 days of storage. Therefore, limonene was dominated by R-limonene when stored for 30 days. Under UV irradiation alone, the ratio of S-linalool decreased from 90 to 85. The ratio of S-linalool decreased from 90 to 87 solely under oxygen exposure. Under UV irradiation–oxygen exposure (UV–O_2_), the ratio of S-linalool decreased from 90 to 86. Although the enantiomer ratio of linalool changed under different storage conditions, S-linalool was still dominant after 30 days of storage. Only under UV irradiation, the ratio of 2R, 5R-linalool oxide decreased from 53 to 49. The ratio of 2R, 5R-linalool oxide decreased from 53 to 50 solely under oxygen exposure, and the ratio of 2R, 5R-linalool oxide decreased from 53 to 49 under UV–O_2_. When stored for 30 days under different storage conditions, although the linalool oxide was dominated by 2S, 5S-linalool oxide, compared with the enantiomeric ratio of the linalool oxide in the original pepper oil, there was little change in the ratio. Under different storage conditions, the enantiomer ratio of carvone changed the same as that of linalol. Although the enantiomer ratio changed, R-carvone was still dominant after 30 days of storage.

In conclusion, the enantiomer ratios of linalol, carvone, and linalool oxide were not significantly changed under different storage conditions, indicating that they had little effect on the flavor change of pepper oil. He et al. [12] found that the enantiomer of linalool in the hard tea beverage was more likely to be transformed at higher storage temperature (45 °C), while pepper oil was stored at 25 °C, which may be unfavorable to the transformation of linalol, carvone, and oxidized linalol enantiomers at this temperature [14]. Under UV–O_2_, limonene was dominated by R-limonene in pepper oil from 20 days after storage, and the proportion of R-limonene gradually increased with the prolongation of storage time. Therefore, the transformation of limonene enantiomers played a certain role in the flavor change of pepper oil.

### 3.2. Changes of Key Aroma Compounds under Different Storage Conditions

**Figure 1:**
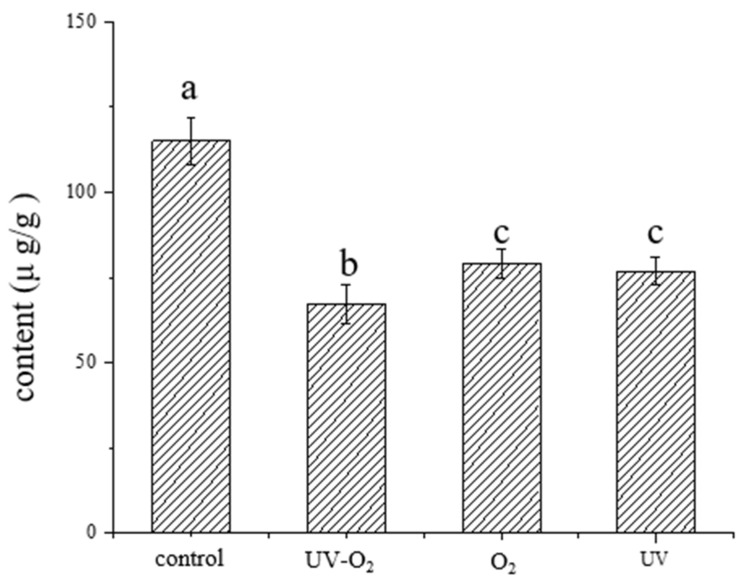
Total content of volatile compounds in fried pepper oil stored for 30 days under different storage conditions. Different letters are significantly different (*p* < 0.05). Tukey’s post hoc test (*p* < 0.05) was performed to compare samples significantly different. Storage conditions: UV (UV irradiation), O_2_ (oxygen exposure), UV–O_2_ (UV irradiation- oxygen exposure); control sample (con); day (d).

It has been reported that many volatiles degrade and oxidize during extraction and storage. For example, monoterpenes in Yuzu (Citrus junos) steam-distilled oil can be converted to monoterpene alcohols and monoterpene oxides [15]. Rouseff et al. [16] found that light and oxygen were the main factors affecting the change of volatile components of citrus essential oils. Our previous study suggested that 1,8-cineole, (E)-2-heptenal, β-myrcene, β-ocimene, limonene, linalool, linalool oxide, and carvone were the key volatile flavor compounds in fried pepper oil [7]. On this basis, the effects of UV irradiation and oxygen exposure storage conditions on the changes of key aroma compounds during the storage period with storage time were investigated. The total contents of volatile compounds in pepper oil stored for 30 days under different storage conditions analyzed by SAFE-GC-MS were shown in Figure 1. The total content of volatile compounds in pepper oil decreased significantly after 30 days of storage, and the total amount under UV–O_2_ storage conditions decreased the most, indicating that UV–O_2_ storage conditions had the greatest impact on the volatile compounds of pepper oil.

**Figure 2:**
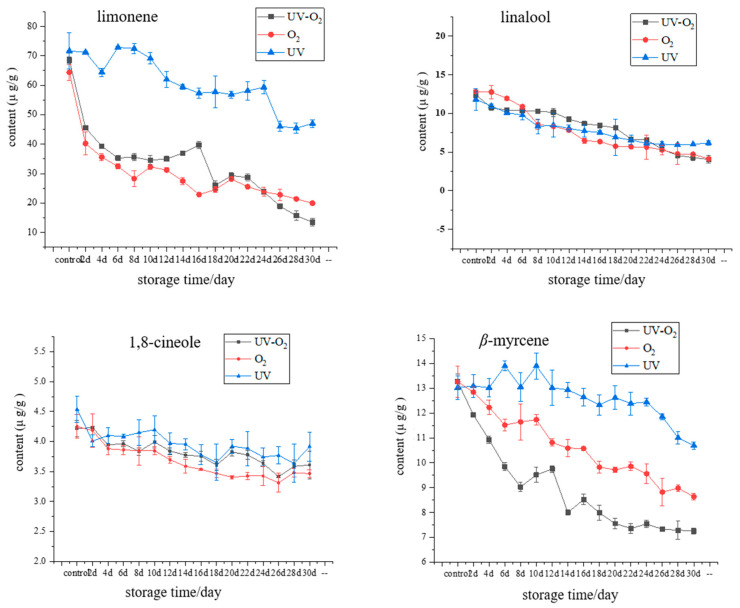

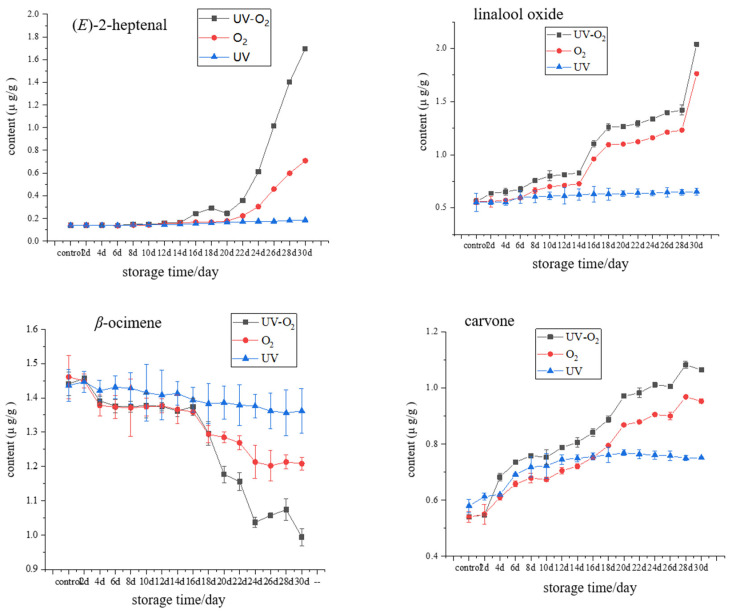
Variation of key aroma compounds in fried pepper oil under different storage conditions.

**Figure 3:**
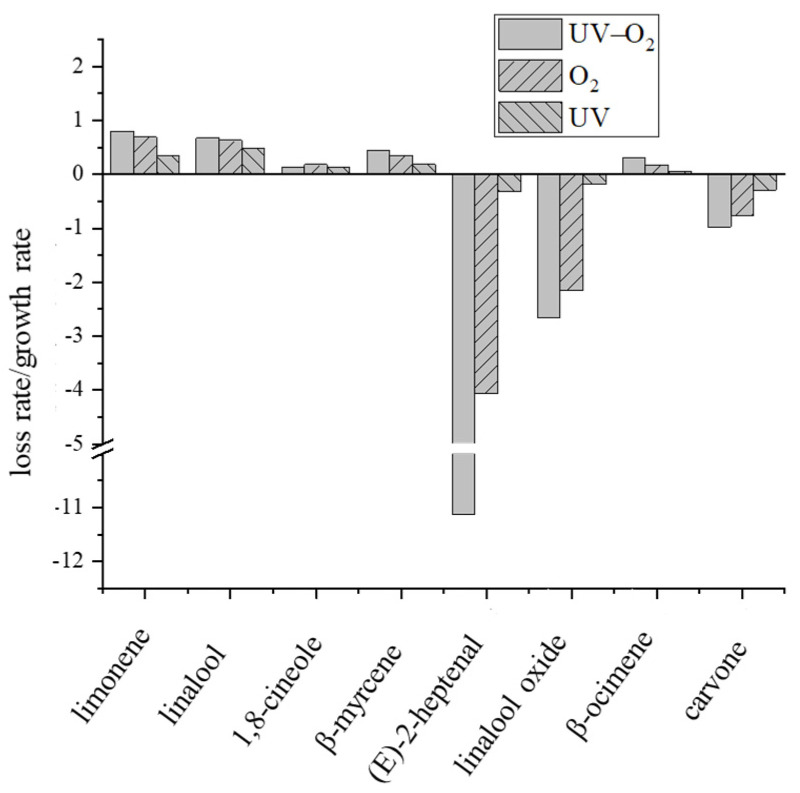
Loss rate/growth rate of key aroma compounds under different storage conditions. Storage conditions: UV (UV irradiation), O_2_ (oxygen exposure), UV–O_2_ (UV irradiation—oxygen exposure); control sample (con); day (d).

The variation of key aroma compounds in pepper oil with storage time under different storage conditions is shown in Figure 2. The contents of limonene, linalool, 1,8-cineole, β-myrcene, and β-ocimene showed a decreasing trend with storage time. Among them, the loss rate of limonene under UV–O_2_ condition was the highest, followed by linalool under UV–O_2_ storage condition (Figure 3), indicating that the combined action of UV and oxygen was more conducive to the degradation or oxidation of limonene and linalool, which were the main compounds that caused flavor attenuation of pepper oil during storage. Previous studies have shown that limonene was readily oxidized in the presence of oxygen. The oxidation of limonene initially leads to the formation of hydroperoxides [17]. As with oxidized unsaturated fatty acids, limonene hydrogen peroxide could be further reacted to produce many products, including limonene oxide, carvone, permaryl acetate, and carvyl acetate [17]. Under acidic conditions, limonene can convert to α-terpineol, which could impart a stale, musty, or piney odor to the product [18]. Limonene can also generate p-cymene through hydrogenation and double bond rearrangement reactions [19]. Baxter et al. [20] showed that linalool could be converted to α-terpenol in a model solution containing aqueous citric acid solution under 24 °C within 20 days. β-Myrcene had the highest loss rate under UV–O_2_ storage conditions, followed by oxygen exposure conditions, which indicated that the oxidation reaction was mainly occurred. Studies have shown that β-myrcene in Yuzu cold-pressed oil can be cyclized to produce γ-terpinene and terpinolene [19]. There was no significant difference in the loss rate of 1,8-cineole under different storage conditions. The loss rate of β-basilene increased rapidly under UV–O_2_ conditions after 16 days of storage. In addition, compared with the aforementioned compounds, the contents of (E)-2-heptenal and linalool oxide showed an increasing trend with storage time. After 20 days of storage, the content of (E)-2-heptenal increased significantly, and the increase rate of (E)-2-heptenal was faster under UV–O_2_ conditions, indicating that UV irradiation promoted the oxidation reaction of linoleic acid. Linalool oxide was the oxidation product of linalool. From 14 days of storage, the growth rate of its content increased significantly, and the effect of UV irradiation was more favorable to the oxidation of linalool. Under UV irradiation conditions, the content of carvone increased gradually during 0–12 days of storage, then tended to balance, and showed an increasing trend under UV–O_2_ and aerobic conditions. Therefore, the combined effect of UV and oxygen was the factor that caused flavor attenuation of pepper oil during storage. Sun et al. [9] compared the effects of sunlight, heat, and oxygen on the aroma degradation of grapefruit essential oil and found that the oily peculiar smell of grapefruit essential oil exposed to UV and oxygen conditions was stronger. This indicated that the combined action of UV radiation and oxygen also caused the aroma of grapefruit essential oil, which was consistent with the results that the combined effect of UV and oxygen was the key factor that caused flavor attenuation of pepper oil during storage.

### 3.3. Investigation of the Volatile Changes under UV–O_2_ Storage Condition

To explore the more accurate changes of the volatile composition under UV–O_2_ storage condition, the pepper oil samples were subjected to GC-MS analysis. A total of 52 compounds were detected in stored pepper oil (Table 3). Compared with the control pepper oil, the types of volatile compounds in the pepper oil stored for 30 days increased by 18, including alcohols, aldehydes, and ketones. Alcohols were derived primarily from the oxidation of aldehydes, such as hexanol, which was an oxidation product of hexanal. However, studies have shown that menthol can be converted from linalool. Linalool was first converted to citronellol by nucleophilic 1,3-transfer (allyl) in acid solution, and then citronellol was converted to menthol by cyclization [21]. Aldehydes and ketones were mainly derived from the oxidative degradation of fats, for example, as (E,E)-2,4-decadienal was the product of linoleic acid oxidation. 6-Methyl-5-heptene-2-ketone exhibited a rotted cabbage leaves odor and was produced in large quantities after 30 days of storage. It had been reported to be the main aromatic volatile in citrus fruits and exhibited remarkable changes in concentration after 25 days of irradiation [22]. In addition, (E)-limonene oxide, p-cymene, α-terpineol, geranyl acetate, and (Z)-p-1,8-menadien-2-ol have increased significantly, which could be the oxidation or degradation products of limonene and linalool. Simon et al. [19] also found that limonene and β-myrcene had been significantly lost during storage at room temperature, whereas p-cymene and monoterpene alcohols increased during the same period. Therefore, the combined UV and oxygen exposure changed the volatile composition of fried pepper oil.

## 4. Conclusions

In summary, the combined UV irradiation and oxygen exposure could cause remarkable aroma deterioration of pepper oil. Chiral compounds in the key aroma compounds of pepper oil include limonene, linalool, linalool oxide, and carvone. R-limonene dominated, and the main structure of other chiral enantiomers remained unchanged after 30 days of storage under UV–O_2_ conditions. The transformation of limonene enantiomers played an important role in the flavor change of pepper oil. The loss or growth rate of key aroma compounds was the largest under UV–O_2_ conditions, and the loss rate of limonene and linalool exhibited the highest under this condition. The aroma attenuation of pepper oil induced by combined UV and oxygen was confirmed to be caused by the degradation and transformation of limonene and linalool.

## Figures and Tables

**Table 1:** Odor description of enantiomers identified in newly prepared fried pepper oil.

Compound	Odor Description [7]
S-(-)-limonene	lemon-like
R-(+)-limonene	orange-like
R-(-)-linalool	woody and lavender-like
S-(+)-linalool	sweet, floral, petitgrain-like
S-(+)-carvone	caraway-like
R-(-)-carvone	spearmint-like
2R, 5R-linalool oxide	floral
2S, 5S-linalool oxide	floral

**Table 2:** Enantiomer ratio of chiral compounds in pepper oil under different storage conditions.

Storage Conditions ^a^	Compound	Enantiomeric Ratio															
		con ^b^	2 d ^c^	4 d	6 d	8 d	10 d	12 d	14 d	16 d	18 d	20 d	22 d	24 d	26 d	28 d	30 d
UV	S-(-)-limonene	60	60	60	59	58	58	55	57	55	55	55	55	53	53	52	52
	R-(+)-limonene	40	40	40	41	42	42	45	43	45	45	45	45	47	47	48	48
	2R, 5R-linalool oxide	53	51	51	51	51	50	51	51	51	51	50	50	50	50	50	49
	2S, 5S-linalool oxide	47	49	49	49	49	50	49	49	49	49	50	50	50	50	50	51
	R-(-)-linalool	10	10	11	11	11	11	11	11	11	12	12	12	12	13	14	15
	S-(+)-linalool	90	90	89	89	89	89	89	89	89	88	88	88	88	87	86	85
	R-(-)-carvone	55	56	56	56	56	56	56	55	55	54	54	54	54	52	52	52
	S-(+)-carvone	45	44	44	44	44	44	44	45	45	46	46	46	46	48	48	48
O_2_	S-(-)-limonene	60	59	57	57	57	55	55	55	54	53	53	52	52	52	51	51
	R-(+)-limonene	40	41	43	43	43	45	45	45	46	47	47	48	48	48	49	49
	2R, 5R-linalool oxide	53	53	53	53	53	52	52	52	52	52	52	52	51	51	51	50
	2S, 5S-linalool oxide	47	47	47	47	47	48	48	48	48	48	48	48	49	49	49	50
	R-(-)-linalool	10	10	11	11	11	11	11	11	11	11	12	12	12	13	13	13
	S-(+)-linalool	90	90	89	89	89	89	89	89	89	89	88	88	88	87	87	87
	R-(-)-carvone	55	54	53	53	53	53	53	52	52	52	52	52	52	52	52	51
	S-(+)-carvone	45	46	47	47	47	47	47	48	48	48	48	48	48	48	48	49
UV–O_2_	S-(-)-limonene	60	58	58	57	55	56	57	55	55	55	54	53	50	50	50	48
	R-(+)-limonene	40	42	42	43	45	44	43	45	45	45	46	47	50	50	50	52
	2R, 5R-linalool oxide	53	53	53	52	52	51	51	51	51	51	50	50	50	50	50	49
	2S, 5S-linalool oxide	47	47	47	48	48	49	49	49	49	49	50	50	50	50	50	51
	R-(-)-linalool	10	10	11	11	11	11	11	12	12	12	12	12	12	13	13	14
	S-(+)-linalool	90	90	89	89	89	89	89	88	88	88	88	88	88	87	87	86
	R-(-)-carvone	55	55	55	55	54	54	54	54	54	54	54	53	53	52	52	51
	S-(+)-carvone	45	45	45	45	46	46	46	46	46	46	46	47	47	48	48	49

^a^ Storage conditions: UV (UV irradiation), O_2_ (oxygen exposure), UV–O_2_ (UV irradiation–oxygen exposure); ^b^ control sample (con); ^c^ day (d).

**Table 3:** Content of volatile compounds of newly prepared fried pepper oil (con) and fried pepper oil stored under UV–O_2_ conditions for 30 days.

Volatile	Concentration (μg/g)		Identification ^b^
	con ^a^	30 Days	
sabinene	0.254 ± 0.04 ^a^	0.245 ± 0.05 ^a^	MS, RI, Std
β-myrcene	13.273 ± 1.03 ^a^	7.259 ± 0.23 ^b^	MS, RI, Std
limonene	68.498 ± 1.42 ^a^	13.456 ± 0.86 ^b^	MS, RI, Std
β-ocimene	1.441 ± 0.05 ^a^	0.994 ± 0.06 ^b^	MS, RI, Std
1,8-cineole	4.213 ± 0.21 ^a^	3.611 ± 0.05 ^b^	MS, RI, Std
δ-3-carene	1.428 ± 0.09 ^a^	2.413 ± 0.09 ^b^	MS, RI, Std
p-cymene	0.294 ± 0.02 ^a^	1.901 ± 0.1 ^b^	MS, RI, Std
terpinolene	0.102 ± 0.01 ^a^	0.196 ± 0.06 ^a^	MS, RI, Std
(E)-limonene oxide	0.342 ± 0.06 ^a^	1.005 ± 0.09 ^b^	MS, RI, Std
1-octen-3-one	n.d.	0.48 ± 0.05	MS, RI, Std
(E)-2-heptenal	0.14 ± 0.01 ^a^	1.697 ± 0.07 ^b^	MS, RI, Std
6-methyl-5-hepten-2-one	n.d.	1.452 ± 0.04	MS, RI, Std
cyclohexanone	n.d.	0.04 ± 0.006	MS, RI, Std
1-hexanol	n.d.	0.552 ± 0.07	MS, RI, Std
(E,Z)-2,6-dimethyl-2,4,6-octatriene	0.524 ± 0.05 ^a^	0.959 ± 0.09 ^b^	MS
octanal	0.23 ± 0.02 ^a^	0.574 ± 0.04 ^b^	MS, RI, Std
(E,E)-2,4-hexadienal	n.d.	0.486 ± 0.03	MS, RI, Std
perillen	0.042 ± 0.001	n.d.	MS, RI, Std
(E)-2-octenal	n.d.	2.916 ± 0.12	MS, RI, Std
1-methyl-4-(1-methylethenyl)-benzene	0.064 ± 0.003 ^a^	0.169 ± 0.03 ^b^	MS, RI, Std
linalool oxide	0.558 ± 0.02 ^a^	2.04 ± 0.08 ^b^	MS, RI, Std
1-octen-3-ol	0.028 ± 0.003 ^a^	1.219 ± 0.14 ^b^	MS, RI, Std
1-heptanol	n.d.	1.19 ± 0.11	MS, RI, Std
acetic acid	0.9 ± 0.04 ^a^	5.808 ± 0.17 ^b^	MS, RI, Std
piperenone	0.036 ± 0.002 ^a^	0.068 ± 0.005 ^a^	MS, RI, Std
(E,E)-2,4-heptadienal	0.208 ± 0.02 ^a^	1.353 ± 0.14 ^b^	MS, RI, Std
4-ethylcyclohexanol	n.d.	0.079 ± 0.006	MS, RI, Std
benzaldehyde	n.d.	0.038 ± 0.002	MS, RI, Std
4-methylcyclohex-3-en-1-one	n.d.	0.245 ± 0.01	MS
menthol	n.d.	0.109 ± 0.007	MS, RI, Std
p-ment-8-en-1-ol	0.066 ± 0.004 ^a^	0.174 ± 0.003 ^b^	MS
linalool	12.414 ± 0.32 ^a^	4.049 ± 0.25 ^b^	MS, RI, Std
linalyl acetate	6.8 ± 0.14 ^a^	2.519 ± 0.13 ^b^	MS, RI, Std
1-octanol	n.d.	0.39 ± 0.09	MS, RI, Std
5-methyl furfural	0.082 ± 0.003	n.d.	MS, RI, Std
caryophyllene	0.048 ± 0.004 ^a^	0.036 ± 0.004 ^a^	MS, RI, Std
6-methyl-3,5-heptadien-2-one	0.03 ± 0.001 ^a^	0.06 ± 0.006 ^a^	MS, RI, Std
dihydrocarvone	n.d.	0.479 ± 0.001	MS
terpinen-4-ol	0.718 ± 0.008	n.d.	MS, RI, Std
5-ethyl-2(5H)-furanone	n.d.	2.122 ± 0.07	MS
(Z)-p-2,8-menadien-1-ol	0.094 ± 0.003 ^a^	0.048 ± 0.006 ^b^	MS
(E)-2-decenal	n.d.	0.574 ± 0.007	MS, RI, Std
(2E,4E)-2,4-decanedienal	n.d.	0.086 ± 0.002	MS, RI, Std
1-nonanol	n.d.	0.185 ± 0.003	MS, RI, Std
(E,E)-2,4-dodecadienal	n.d.	0.087 ± 0.001	MS, RI, Std
terpinyl acetate	0.316 ± 0.06 ^a^	0.38 ± 0.006 ^a^	MS, RI, Std
α-terpineol	0.262 ± 0.008 ^a^	0.352 ± 0.007 ^b^	MS, RI, Std
piperitone	0.052 ± 0.002 ^a^	0.055 ± 0.002 ^a^	MS, RI, Std
carvone	0.54 ± 0.007 ^a^	1.06 ± 0.049 ^b^	MS, RI, Std
neryl acetate	0.078 ± 0.001 ^a^	0.15 ± 0.003 ^b^	MS, RI, Std
6-ethenyltetrahydro-2,2,6-trimethyl-2H-pyran-3-ol	0.086 ± 0.002 ^a^	0.236 ± 0.003 ^b^	MS
(Z)-p-1,8-menadien-2-ol	0.082 ± 0.007 ^a^	0.264 ± 0.006 ^b^	MS
(E)-carveol	0.146 ± 0.003 ^a^	0.046 ± 0.001 ^b^	MS, RI, Std
2-(4-methylphenyl)propan-2-ol	0.022 ± 0.002	n.d.	MS
(Z)-carveol	0.082 ± 0.001 ^a^	0.041 ± 0.001 ^b^	MS, RI, Std
2-acetylpyrrole	0.026 ± 0.001 ^a^	0.065 ± 0.002 ^b^	MS, RI, Std

^a^ control sample (con). ^b^ Identification based on Nist 14 mass spectral database (MS); published RIs; confirmed by authentic standards (Std). Means within different letters are significantly (*p* < 0.05) different for the same parameter. Tukey’s post hoc test (*p* < 0.05) was performed to compare significantly different means and samples.

## Data Availability

Not applicable.
